# Hypoglossal Nerve Palsy as a Cause of Severe Dysphagia along with the Oropharyngeal Stenosis due to Occipitocervical Kyphosis

**DOI:** 10.1155/2019/7982847

**Published:** 2019-03-06

**Authors:** Tomohiro Watanabe, Masato Anno, Yoshitaka Matsubayashi, Yuki Nagasako, Kaori Sakuishi, Yoh Fujimoto, Naohiro Tachibana, Yuki Taniguchi, Toshihiro Hayashi, Yasushi Oshima, Shoji Tsuji, Sakae Tanaka

**Affiliations:** ^1^Department of Orthopaedic Surgery, The University of Tokyo Hospital, Japan; ^2^Department of Neurology, The University of Tokyo Hospital, Japan

## Abstract

Hypoglossal nerve palsy (HNP) is a potential cause of dysphagia. A 66-year-old man presented to our hospital with dysphagia and neck pain. One year prior to his first visit, he had been diagnosed with upper cervical tuberculosis and had undergone posterior C1-2 fixation. The physical examination led to the diagnosis of dysphagia with HNP, and he had severe weight loss. Radiographic examination revealed that the O-C kyphosis had been exacerbated and that the deformity was likely the primary cause of HNP. To restore the swallowing function, O-C fusion surgery was performed. Postoperatively, the patient showed immediate improvement of dysphagia with gradual recovery of hypoglossal nerve function. In the last follow-up evaluation, swallowing function was confirmed with no signs of HNP. Our results indicate that HNP could be more prevalent in cases with severe cervical kyphosis, being underdiagnosed due to the more apparent signs of the oropharyngeal narrowing.

## 1. Introduction

Hypoglossal nerve palsy (HNP) is a rare condition that impairs tongue movement, which can result in dysphagia. To our knowledge, there have been only limited reports of HNP associated with spinal diseases, and therefore, the etiology remains unclear. In the present study, we report a case of HNP which occurred after postoperative occiput-cervical (O-C) kyphosis in conjunction with the oropharyngeal narrowing and which was improved by correcting the deformity.

## 2. Case Report

### 2.1. History

A 66-year-old man had been diagnosed with infectious cervical tuberculosis on C1 and undergone posterior C1-2 screw-plate fixation at a hospital in India one year prior to his visit to our hospital. Although the surgery was successful and his neck pain had improved, his swallowing function had gradually worsened over the nine-month period after the initial surgery, along with loss of reduction. Due to progressive dysphagia and severe weight loss, he was referred to our hospital. His medical history included hypertension and mild diabetes mellitus (HbA1c 6.2% NGSP). He had been given antitubercular treatment since he was diagnosed with infectious cervical tuberculosis at the local hospital.

### 2.2. Examination

The patient's height was 165 cm, his weight was 52 kg (BMI 19), and he exhibited normal cognitive function. He had lost 25 kg over 7 months because of difficulty in swallowing, and a nasogastric (NG) tube was placed for tube feeding. Neurological examination of the patient revealed left dominant proximal arm muscle weakness with atrophy, dysesthesia in distal fingers, hyperreflexia throughout with bilateral extensor plantar reflex. Oral examination was remarkable for left tongue atrophy as well as left tongue deviation, which was consistent with unilateral HNP. Routine blood work showed slightly elevated level of C reactive protein (CRP), but the findings were otherwise normal.

**Figure 1:**
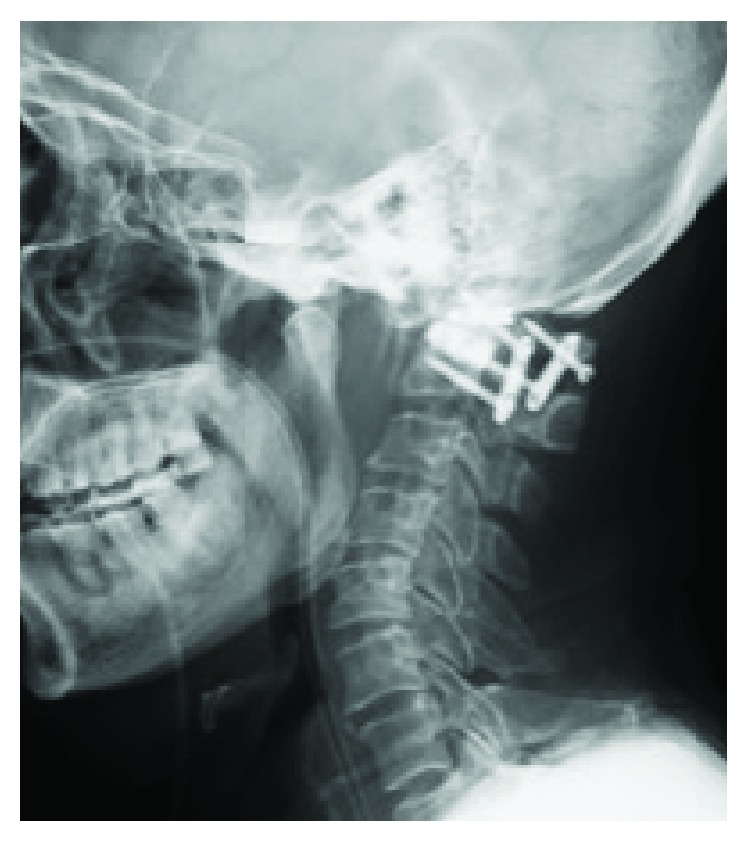
Lateral cervical X-ray at the first visit to our hospital showing instruments on C1/C2, O-C2 angle of 17-degree kyphosis, and vertical subluxation: Redlund-Johnell's distance, 22 mm. Space available for the cord (SAC), atlantodental interval (ADI), and C2-7 angle are 15 mm, 2 mm, and 33°, respectively.

**Figure 2:**
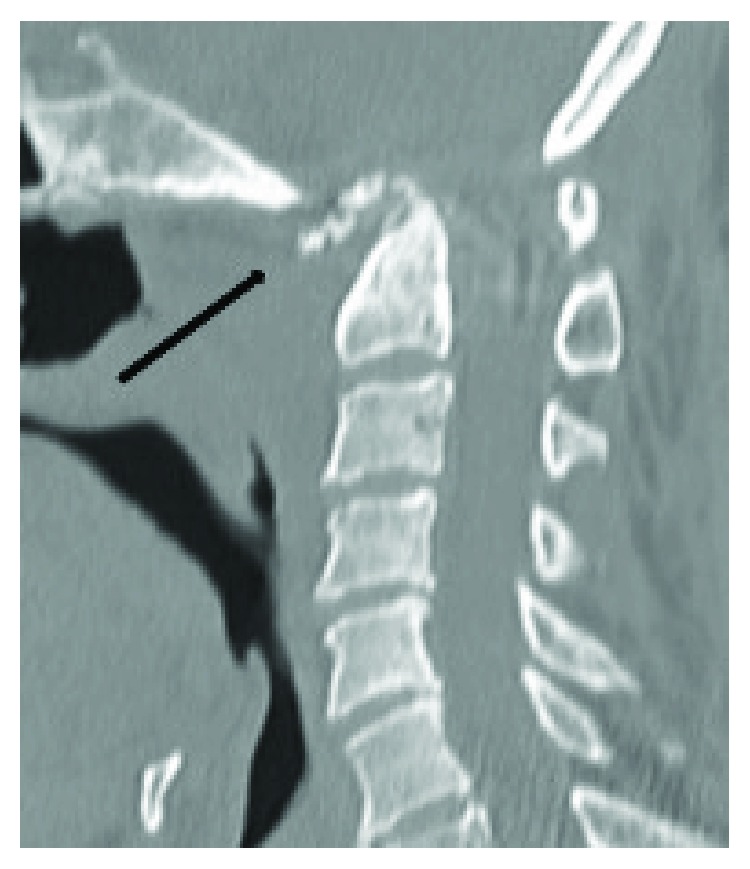
Computed tomography (CT) of the cervical spine showing erosion of anterior arch of C1 vertebra and dens (black arrow).

**Figure 3:**
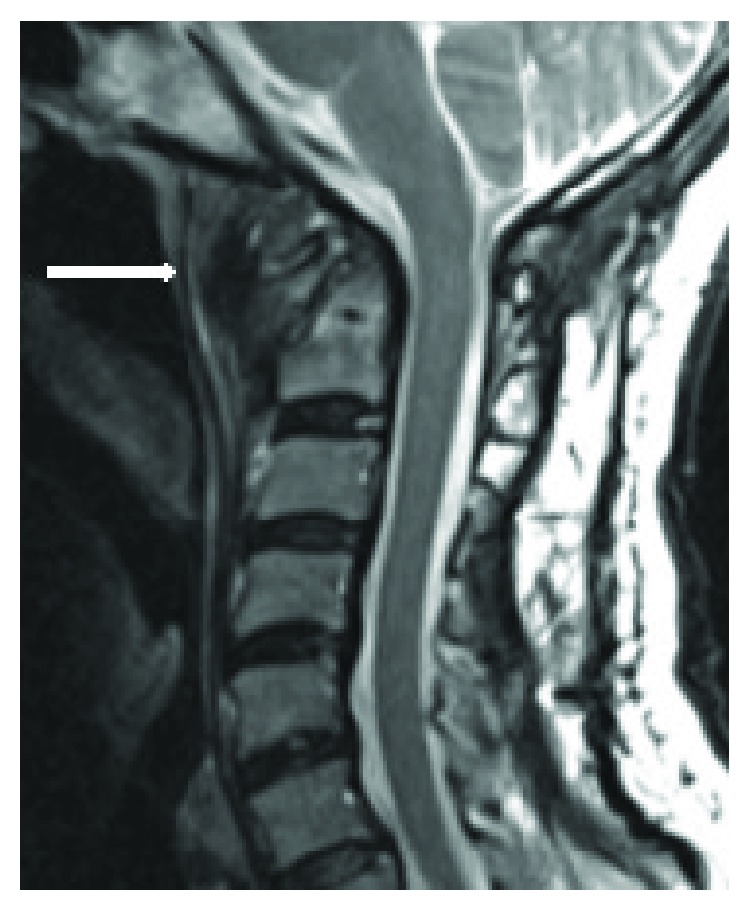
Sagittal T2-weighted image of magnetic resonance imaging of the cervical spine at the first visit showing a mass on retropharyngeal space with heterogeneous intensity (white arrow).

Chest X-ray results showed no specific abnormality. Lateral cervical X-ray showed O-C2 angle of 17-degree kyphosis (Figure 1). Computed tomography (CT) showed an erosive lesion at dens and anterior arch of the atlas (Figure 2). Magnetic resonance imaging showed a space-occupying lesion in the retropharyngeal space, which presented with heterogeneous signals on both T1- and T2-weighted images (Figure 3). Further sequential review of previous imaging studies revealed that, contrary to the progression of O-C kyphosis, the lesion had been gradually decreasing in size.

In sum, the patient had two main problems: severe dysphagia and subsequent malnutrition and neck pain. Initially, we assumed that the dysphagia was primarily caused by oropharyngeal stenosis resulting from O-C kyphosis [1–3]. However, since no findings of the intracranial pathology were observed and the patient exhibited a persistent unilateral HNP and severe swallowing dysfunction, we eventually hypothesized that the dysphagia would have been deteriorated even worse due to the limited tongue movement. In other words, both O-C kyphosis and HNP were related to dysphagia, and it was not possible to draw a clear line by which these two factors were divided regarding the etiology. No conservative treatments had improved these symptoms, and we therefore decided to perform a corrective surgery to restore the swallowing function and to relieve the neck pain.

### 2.3. Operation

**Figure 4:**
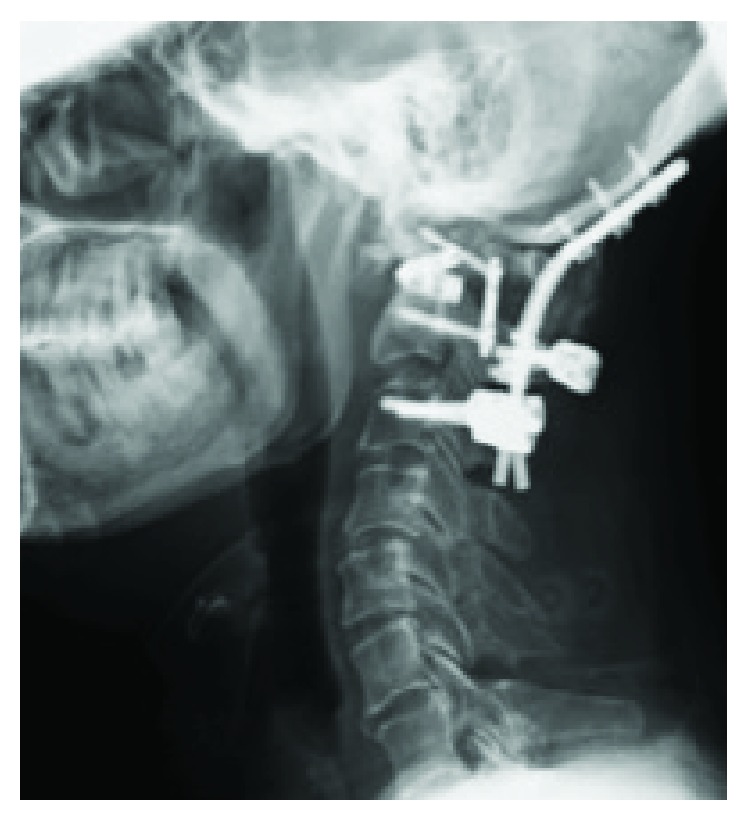
Lateral cervical X-ray after the posterior O-C3 fusion surgery at our hospital showing O-C2 angle of 6-degree lordosis.

Posterior O-C3 fusion surgery with iliac bone graft was performed without complications. The O-C kyphosis was corrected to 6-degree lordosis on O-C2 (Figure 4). Findings of the tissue biopsy from the retropharyngeal mass were negative for infectious etiology. The postoperative course was uneventful. His swallowing function concomitantly with his tongue movement improved in two weeks after the surgery. At the final follow-up visit at five months, bone union was observed, and swallowing function was confirmed without further deterioration.

## 3. Discussion

The hypoglossal nerve or the twelfth cranial nerve is a pure motor nerve which controls both the intrinsic and extrinsic muscle of the tongue. Similar to the other cranial nerves, it is divided into three sections: supranuclear, nuclear, and infranuclear. Knowing how the tongue movement and coordination are affected allows clinicians to narrow down the cause of HNP [4]. For instance, supranuclear lesions usually produce weakness of the contralateral side of the tongue, while nuclear or infranuclear pathology develops dysfunction of the hypoglossal nerve of the involved side [5, 6], which eventually predisposes patients to tongue atrophy, deviation, and dysphagia. The hypoglossal nerve is also divided into four to five segments based on its anatomical features [4, 7]. The nerve arises from its nucleus and exits the skull base through the hypoglossal foramen. The extracranial part runs lateral to the carotid artery and anterior to the upper cervical spine, and it finally innervates the tongue [8–10]. The nerve can be damaged at every section in its trajectory. Specifically, one study showed imaging features of the hypoglossal nerve by dividing it into four segments—intra-axial, cisternal, skull base, and extracranial segments—and identified pathologies for each segment: vascular, neoplasia, infection/inflammation, trauma, and autoimmune [4].

The etiologies of HNP in relation to spinal diseases can be categorized as follows: direct injury [11–14], indirect mechanical extension [13–19], insufficient circulation [20, 21], and inflammation [22, 23]; each occurs at the extracranial segment. First, direct injury to the spine has been shown to cause HNP. In our case, imaging studies did not show any evidence of direct injury to the lower brainstem or cervical spinal cord. An enlargement of the mass on the retropharyngeal space was initially observed, but it gradually decreased in size after the first surgery. Furthermore, no specific findings suggested the recurrence of infection since he came to us. Therefore, it was not likely that inflammation in relation to the retropharyngeal mass was the primary cause of HNP. Taking all these conditions into consideration, we hypothesized that the mechanical extension due to hyperflexion of the cervical spine played a major role in our case.

Studies have shown that O-C kyphosis can result in oropharyngeal stenosis. Specifically, some authors have demonstrated the usefulness of O-C2 angle as a predictor of postoperative dysphagia and/or dyspnea [2, 3]. Izeki et al. argued that O-C2 angle at least more than preoperative neutral position is needed to avoid persistent dysphagia [1]. These studies suggest that the prominent cause of dysphagia in our case was the upper cervical kyphosis. However, it was also of note that the patient had persistent unilateral HNP, and the dysphagia was so severe that the patient was not capable of any oral intake, which finally made us assume that HNP was another critical cause of restricted swallowing function. Given that some previous case reports have argued that HNP is associated with hyperflexion of the neck [17–19] and the considerable number of cases with dysphagia due to the O-C2 kyphosis, the authors suggest that HNP could be more prevalent in such deformity cases, being masked by the more apparent signs of the oropharyngeal stenosis.

**Figure 5:**
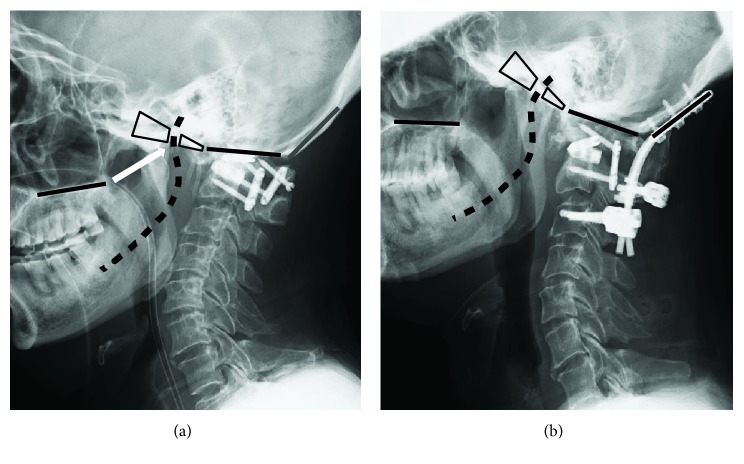
(a) Pre- and (b) postoperative lateral cervical X-rays showing the surgical plan for the change of direction of hypoglossal nerve (black dotted lines). The white arrow points the place at which the nerve exits from the hypoglossal foramen and is stretched backward due to the O-C kyphosis.

In the current case, after we reduced the malalignment in the O-C junction, not only did he experience remarkable improvement in his swallowing function but also gradual restoration of his tongue movement. We therefore propose that the recovery from HNP can be primarily explained by the surgical release of the mechanical extension force of the hypoglossal nerve (Figure 5).

In conclusion, these findings suggest that HNP may be more prevalent in cases with O-C kyphosis, lurking behind the more obvious signs of the oropharyngeal narrowing. Additionally, corrective surgery can be a reasonable treatment, not just to improve the swallowing function but also to restore the hypoglossal nerve function.
